# Photonic bandgap of inverse opals prepared from core-shell spheres

**DOI:** 10.1186/1556-276X-7-457

**Published:** 2012-08-15

**Authors:** Bo-Tau Liu, Ya-Li Lin, Shao-Xian Huang

**Affiliations:** 1 Department of Chemical and Materials Engineering, National Yunlin University of Science and Technology, 123 Univ. Rd., Sec. 3, Douliou, Yunlin, 64002, ,Taiwan, Republic of China

**Keywords:** Core-shell, Shrinkage, Photonic crystal, Inverse opal, Polystyrene

## Abstract

In this study, we synthesized monodispersed polystyrene (PS)-silica core-shell spheres with various shell thicknesses for the fabrication of photonic crystals. The shell thickness of the spheres was controlled by various additions of tetraethyl orthosilicate during the shell growth process. The shrinkage ratio of the inverse opal photonic crystals prepared from the core-shell spheres was significantly reduced from 14.7% to within 3%. We suspected that the improvement resulted from the confinement of silica shell to the contraction of PS space during calcination. Due to the shell effect, the inverse opals prepared from the core-shell spheres have higher filling fraction and larger wavelength of stop band maximum.

## Background

Based on the periodic dielectric structure, artificial photonic crystals exhibit a photonic bandgap caused by Bragg diffraction [1,2]. The special optical properties have been widely explored for various applications including optical waveguides, sensors, light filters, optical integrated circuits, and low-threshold telecommunication lasers [3-7]. Especially, an inverse opal structure is more desirous due to the occurrence of a complete photonic bandgap, in which incident wavelengths are forbidden for every state of polarization and propagation direction [8,9]. The face-centered cubic (FCC) array spheres are usually used as the templates for the preparation of inverse opals: filling the high-refractive-index materials into the voids among the spheres, and then removing the spheres by calcinating or washing with organic solvents [10,11]. The calcination method, often used in the reported studies, is more effective in removing the organic polymeric spheres than washing with organic solvents. Nevertheless, a 10% to 20% shrinkage is inevitable [12,13]. The drawback leads to difficultly fabricate the inverse opals with the wavelength of stop band maximum (*λ*_m_) and the corresponding photonic bandgap as designed.

The photonic bandgap of photonic crystals depends mainly on lattice constant (or diameter of spheres), refractive indices of components (spheres and voids), and filling fraction. The filling fraction of spheres for the self-assembled photonic crystals, featuring a close-packed FCC structure, is 0.74, which is impossible to change for the self-assembly approach. In most cases of inverse opals, only few materials such as silica and titania can be used to fill up the voids among air spheres due to the issues of material properties and preparation processes [14,15]. Hence, the selectivity of refractive indices of components of inverse opals is limited. As a result, the photonic bandgap is usually not controlled by the refractive indices. A simple way to control the photonic bandgap is to change the size of spheres. However, *λ*_m_ is difficultly predicted due to the shrinkage caused by calcination as aforementioned.

Core-shell or hollow colloidal photonic crystals have revealed special optical properties and chemical sensing as reported elsewhere [7,16-18]. In this study, we built upon a core-shell colloidal technique to fabricate inverse opals. The position of *λ*_m_ can be predicted due to the low shrinkage ratio and controlled by the variation of shell thickness.

## Methods

### Materials

Styrene (Sigma-Aldrich, St. Louis, MO, USA) was purified by distillation under reduced pressure and then stored in a refrigerator. Poly(vinylpyrrolidone) (PVP K30, Sigma-Aldrich), 2,2′-azobis(2-methylpropionamidine) dihydrochloride (AIBA, Sigma-Aldrich), tetraethyl orthosilicate (TEOS, Sigma-Aldrich), ammonium hydroxide solution (33%, Sigma-Aldrich), hydrochloric acid (36%, Katayama Chemical, Osaka, Japan), and ethanol (99.8%, Panreac, Castellar del Vallès, Spain) were used as received. Deionized water (DI water, >18 MΩ·cm) was used in all experiments.

### Synthesis and core-shell PS-silica spheres

Polystyrene (PS) spheres were synthesized by emulsifier-free emulsion polymerization according to the modification of the previous reports [16,19]. Briefly, 5 g of styrene and 50 g of DI water were mixed in a reaction vessel. After purging with nitrogen for at least 30 min, the vessel was placed in an oil bath at 70°C under nitrogen atmosphere. Then, 0.75 g of PVP K30 was added into the vessel as a stabilizer, and 0.13 g of AIBA was added to the mixture to initiate the polymerization reaction. The reaction was carried out for 24 h at a stirring rate of 750 rpm.

The as-prepared PS spheres were washed with DI water by centrifugation to remove the residual initiator and unreacted monomers. To prepare the PS-silica core-shell spheres, 0.55 g of the PS spheres and 4 g of ammonia were mixed with 74 g of ethanol at 55°C. TEOS was then added dropwise into the solution. Next, the mixture was stirred at a rate of 350 rpm over 8 h for the sol–gel reaction [20,21].

### Preparation of photonic crystals

Glass substrates were immersed in piranha solution (H_2_SO_4_/H_2_O_2_, 7:3, *v*/*v*) for 30 min and then washed with DI water. The washed glass was put horizontally into a beaker containing 0.5 wt.% PS-silica core-shell sphere solution, which was placed in a water bath at 45°C. Opal photonic crystals were gradually formed while the water in the solution evaporated. The inverse opal photonic crystals were prepared by filling the void spaces among the sphere array with a silica sol–gel precursor. Briefly, the as-prepared opal photonic crystal sample was vertically dipped in a vessel containing the mixture of TEOS, ethanol, and 0.2 M HCl. The sample was raised from the vessel, and then dried at 100°C for 40 min. The dipping process was repeated to increase infiltration. Finally, the sample was heated to 350°C, held for 2 h, re-heated to 500°C, and then held for 8 h.

### Characterization

The morphologies of the PS-silica core-shell spheres were observed from scanning electron microscopy (SEM) images using a field-emission scanning electron microscope (JSM-7401F, JEOL, Akishima-shi, Japan) and transmission electron microscopy (TEM) images using a high-resolution transmission electron microscope (JEM-2010, JEOL). The optical properties of the photonic crystals were measured using an ultraviolet–visible (UV–vis) spectrophotometer (Lambda 850, PerkinElmer, Waltham, MA, USA).

## Results and discussion

**Figure 1:**
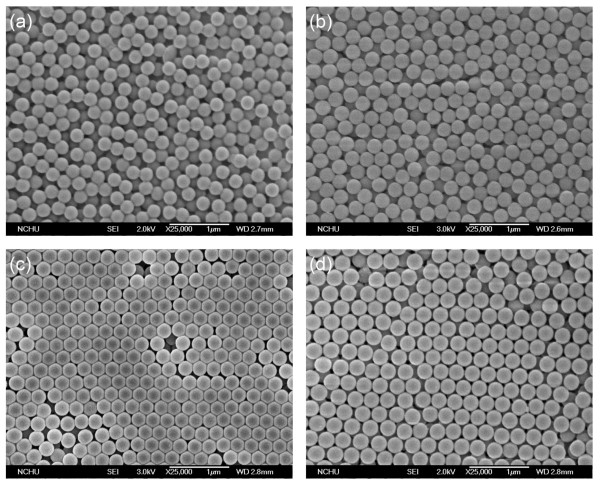
**SEM images of the pristine PS spheres and the PS-silica core-shell spheres.** (**a**) PC0, (**b**) PC3, (**c**) PC6, and (**d**) PC9.

**Figure 2:**
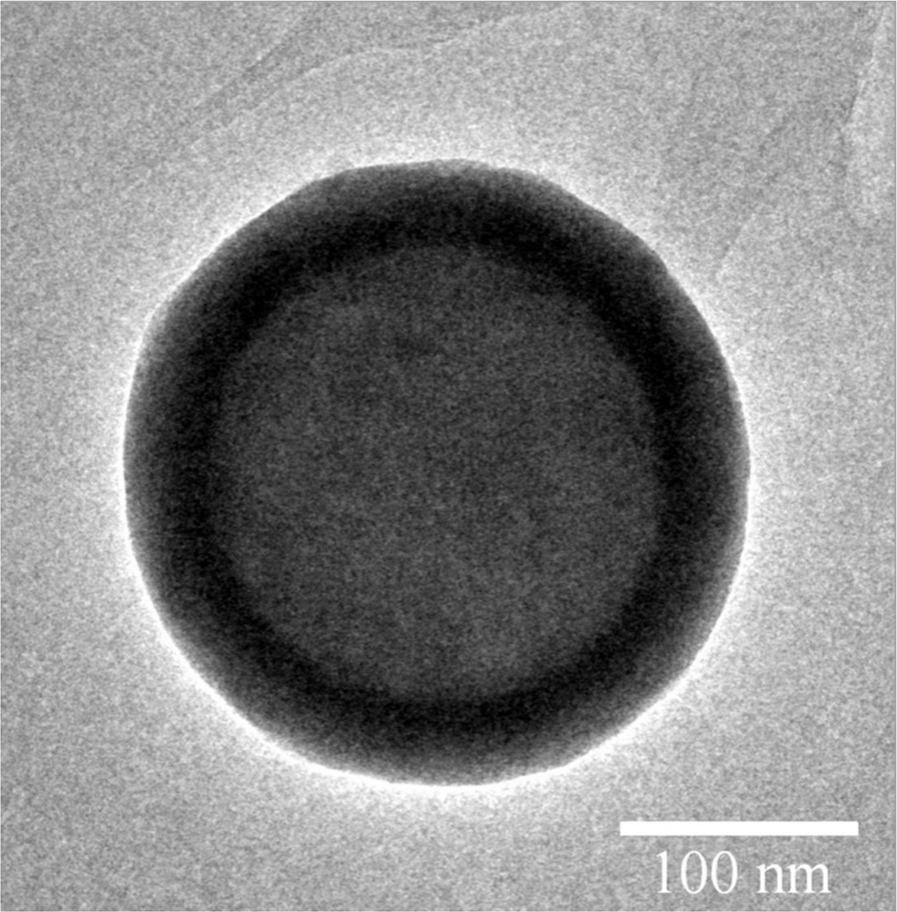
TEM image of the PS-silica core-shell spheres (PC9).

Figure 1 shows the SEM images of the as-prepared PS particles and the PS-silica core-shell particles with various shell thicknesses. Regardless of monotonic or core-shell structures, the particles reveal a nearly spherical shape and monodispersed distribution. As shown in Figure 1 and Table 1, the particle size becomes larger with the increasing TEOS concentration. Because the diameter of the PS core particles was not changed, this result implies that the shell thickness increases while the TEOS concentration was raised. The core-shell structure was also confirmed by the TEM image as shown in Figure 2.

**Table 1:** Compositions of PS-silica core-shell spheres and properties of the corresponding photonic crystals

**Code**	**TEOS (M)**	**Opal**			**Inverse opal**		
		***λ***_**m**_^**a **^**(nm)**	***d***_**m**_^**b **^**(shell thickness) (nm)**	***d***_**s**_^**c **^**(shell thickness) (nm)**	***λ***_**m**_^**d **^**(nm)**	***d***_**a**_^**e **^**(nm)**	**Shrinkage ratio**^**f **^**(%)**
PC0	0	530	224 ± 2.9 (0)	223.4 (0)	348	190.5	14.7
PC3	0.03	564	242 ± 2.6 (20)	241.2 (17.8)	428	200.6	10.2
PC6	0.06	586	250 ± 4.2 (26)	252.4 (29.0)	501	220.6	1.3
PC9	0.09	606	262 ± 3.2 (38)	262.5 (39.1)	528	218.3	2.3

^a^Wavelength of stop band maximum for the opal structure, observed from Figure 3; ^b^diameter of core-shell spheres, measured from SEM images; ^c^diameter of core-shell spheres, calculated from Equation 1; ^d^wavelength of stop band maximum for the inverse opals, measured from the UV–vis spectra; ^e^diameter of air spheres, calculated from Equation 1, assuming that the thickness of the shell was fixed; ^f^estimated from the ratio of *d*_a_ to the pristine diameter of the PS spheres.

**Figure 3:**
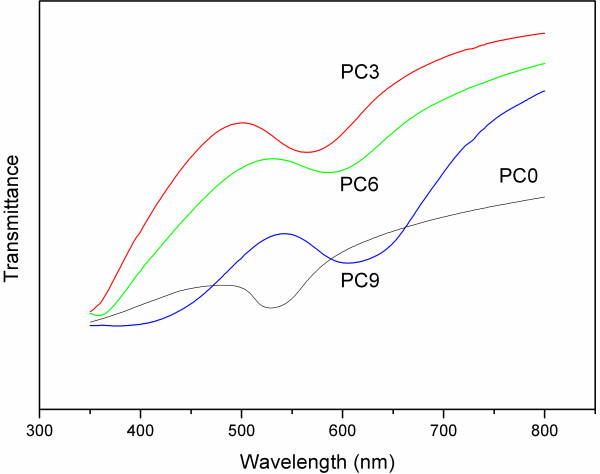
Transmission spectra of the photonic crystals prepared from core-shell PS-silica spheres with various shell thicknesses.

Figure 3 shows the transmission spectra of the opal photonic crystals prepared from the core-shell spheres. The corresponding *λ*_m_ has a red shift with the increase of TEOS concentration, indicating the increase of the particle size. The position of *λ*_m_ of photonic crystals with the FCC crystal structure can be estimated using a modified form of Bragg’s law with respect to normal incidence:

(1) $$\lambda_{\text{m}}=1.155\cdot a\cdot \sqrt{0.24\cdot {n_{\text{v}}}^{2}+0.74\cdot \left(f\cdot {n_{\text{sh}}}^{2}+\left(1-f\right)\cdot {n_{\text{c}}}^{2}\right)}\text{,}$$

where *a* is the lattice constant; *f* is the volume fraction of the silica shell in the core-shell spheres; and *n*_v_, *n*_sh_, and *n*_c_ are the refractive indices of the voids, the shell, and the core, respectively. Estimated from Equation 1 and Figure 3, the average particle size and the shell thickness of the core-shell spheres were shown in Table 1, which are in good agreement with the observation from electronic microscopy, within a difference of 3 nm.

**Figure 4:**
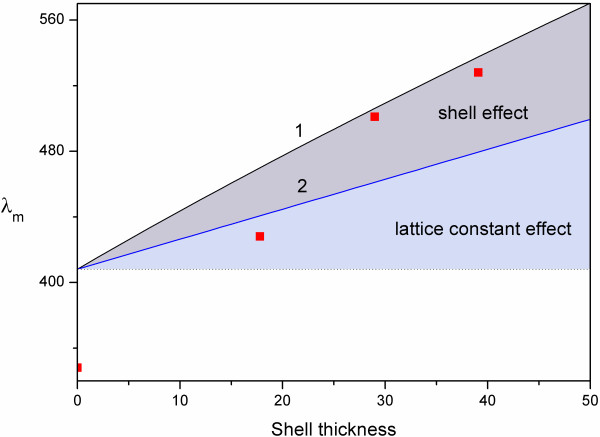
**Wavelength of stop band maximum for the inverse opals as a function of shell thickness.** Curve 1: calculated from Equation 1; curve 2: calculated from Equation 1 but ignoring the shell filling effect; red square: measured from UV–vis spectra.

The *λ*_m_ of the corresponding inverse opals are shown in Figure 4 and Table 1. The shrinkage of air spheres in the inverse opal caused by the calcination was evaluated. Observing from Table 1, without silica shell, the shrinkage ratio of air spheres reached as high as 14.7%, close to the value reported elsewhere [12,13]. However, with the increase of the silica shell thickness, the shrinkage decreased significantly to within 3%. The improvement may result from the fact that the silica shell confined the PS space to avoid the shrinkage arising from sol–gel condensation during the high-temperature calcination.

According to Equation 1, the shift of *λ*_m_ may arise from the lattice constant and the filling volume fraction. Therefore, the shift of *λ*_m_ of the inverse opals prepared from the core-shell spheres was affected by lattice constant effect and shell effect, shown in Figure 4. On the same lattice constant, the inverse opals prepared from the core-shell spheres can result in the larger shift of *λ*_m_ (curve 1 in Figure 4) than the inversed opals with connective air spheres (curve 2 in Figure 4) due to the shell effect (the higher filling fraction). Moreover, on the same *λ*_m_, the inverse opals made by the core-shell technique have smaller air spheres and higher filling fraction than those prepared by the traditional method (not core-shell structure). The improvement may result in a stronger structure. Observing from Figure 4, the position of *λ*_m_ is very close to the predicted point while the shell thickness is larger than 29 nm.

## Conclusions

We have prepared monodispersed PS-silica core-shell spheres with various shell thicknesses for the preparation of photonic crystals. With the shell protection, the shrinkage ratio with respect to the inverse opals can be significantly reduced from 14.7% to within 3%. The inverse opals prepared from the core-shell spheres have smaller air spheres and higher filling fraction than those with connective air spheres, resulting in a stronger structure.

## Abbreviations

AIBA: 2,2′-azobis(2-methylpropionamidine) dihydrochloride; DI water: deionized water; FCC: face-centered cubic; PS: polystyrene; PVP: poly(vinylpyrrolidone); TEOS: tetraethyl orthosilicate.

## Competing interests

The authors declare that they have no competing interests.

## Authors’ contributions

The manuscript was written through the contributions of all authors. BTL conceived of the study, carried out the experimental analyses, and revised the manuscript. YLL performed the most syntheses and characterizations. SXH helped in the sample tests. All authors read and approved the final manuscript.

## Authors’ information

BTL is currently an associate professor in Yuntech, Taiwan. YLL and SXH are graduate students in Yuntech under the supervision of BTL.
